# Impact of acupuncture on lower limb motor function recovery after stroke: A systematic review and meta-analysis

**DOI:** 10.1097/MD.0000000000050712

**Published:** 2026-10-02

**Authors:** Yaheng Zhao, Xiaochuan Zhu, Lei Xie, Dongyi Yang, Jiabin Wang, Yangyang Liu, Tao Wu, Renyi Qian

**Affiliations:** a Neurology Department, First Affiliated Hospital of Henan University of Chinese Medicine, Zhengzhou, Henan Province, China.

**Keywords:** acupuncture, Berg Balance Scale, Fugl-Meyer Assessment Lower Extremity, lower extremity, Modified Barthel Index, rehabilitation, stroke

## Abstract

**Background:**

Lower-extremity motor impairment after stroke is a major determinant of mobility limitation and persistent disability. Acupuncture is frequently used as an adjunct to conventional rehabilitation, but the magnitude and consistency of its effects on lower-limb recovery remain uncertain.

**Methods:**

This systematic review and meta-analysis followed the Preferred Reporting Items for Systematic Reviews and Meta-Analyses statement. PubMed, Embase, Web of Science, and the Cochrane Library were searched from inception to December 31, 2025, without language restrictions. Randomized controlled trials evaluating needle-based acupuncture for poststroke lower-limb recovery were included. Risk of bias was assessed using the Cochrane Risk of Bias tool for randomized trials version 2.0, and the certainty of evidence for the main outcomes was evaluated using the Grading of Recommendations Assessment, Development and Evaluation approach. Meta-analyses were conducted in Stata 18.0 using random-effects models, and treatment effects for continuous outcomes were pooled as standardized mean differences (SMDs) with Hedges *g* correction and 95% confidence intervals (CIs). Because fewer than 10 studies contributed to each outcome, formal funnel-plot asymmetry tests, including Egger test, were not performed; publication bias was considered qualitatively.

**Results:**

Six trials met the inclusion criteria. Acupuncture was associated with improved lower-extremity motor function, as measured by the Fugl-Meyer Assessment Lower Extremity (SMD = 0.51, 95% CI = 0.32–0.71; *I*^2^ = 53.9%; low certainty). Balance assessed by the Berg Balance Scale also favored acupuncture but showed substantial heterogeneity (SMD = 0.96, 95% CI = 0.35–1.56; *I*^2^ = 91.5%; very low certainty). The Modified Barthel Index did not demonstrate a statistically significant benefit (SMD = 0.26, 95% CI = 0.16–0.68; *I*^2^ = 86.3%; very low certainty). The high heterogeneity for Berg Balance Scale and Modified Barthel Index indicates that these pooled estimates should be interpreted cautiously.

**Conclusion:**

Acupuncture may provide low-certainty incremental improvements in poststroke lower-extremity motor impairment. Evidence for balance and activities of daily living remains very uncertain because of substantial heterogeneity, limited sample size, and variability in acupuncture protocols and control conditions. Larger, prospectively registered, methodologically rigorous trials with standardized protocols, comparable rehabilitation dose, and clinically interpretable outcome reporting are needed.

## 1. Introduction

Stroke remains a leading cause of death and long-term adult disability. According to the World Health Organization, stroke was the third leading cause of death and disability globally in 2021, with an estimated 93.8 million prevalent cases and 11.9 million new cases; the global stroke burden measured by disability-adjusted life years increased from 137 million in 2000 to 160 million in 2021. Lower-extremity motor impairment is particularly important because mobility recovery after stroke depends on regaining lower-limb function, sitting and standing balance, and walking ability. Approximately two-thirds of patients initially experience impaired mobility after stroke.^[1]^ Consensus-based recommendations for stroke recovery research further emphasize that lower-extremity function, balance, and mobility are core constructs for poststroke rehabilitation outcomes, and prospective cohort evidence suggests that 80% to 95% of stroke survivors regain walking independence within the first 3 to 6 months, whereas this proportion may decrease to about 60% among those unable to walk in the first week after stroke.^[2]^ These data provide a quantitative rationale for focusing specifically on lower-limb recovery rather than overall motor recovery alone.

Lower-extremity deficits directly constrain standing, balance control, transfers, gait, community ambulation, and independence in activities of daily living, while also increasing fall risk. Contemporary stroke rehabilitation guidance emphasizes task-specific, intensive, and progressive mobility training, yet many patients experience incomplete restoration of gait and balance despite structured multidisciplinary care. From a mechanistic perspective, acupuncture may activate peripheral somatosensory afferents and influence central motor recovery through modulation of corticospinal excitability, interregional functional connectivity, sensorimotor network reorganization, spasticity-related neuromuscular control, and neuroinflammatory or neurotrophic signaling related to postischemic neuroplasticity.^[3,4]^ Although these mechanisms remain exploratory and cannot establish clinical efficacy by themselves, they provide a biologically plausible rationale for evaluating acupuncture as an adjunct to conventional poststroke lower-limb rehabilitation.

Several recent evidence syntheses have evaluated acupuncture for poststroke motor outcomes, but important limitations remain for informing lower-extremity–specific decision-making. A 2024 systematic review and network meta-analysis compared acupuncture modalities for poststroke lower-limb motor dysfunction and suggested potential differential effectiveness across approaches, while also highlighting variability in evidence quality and the need for high-quality trials.^[5]^ In addition, gait-parameter–focused meta-analytic evidence indicates potential benefits in spatiotemporal gait metrics when acupuncture is added to rehabilitation, although heterogeneity in participant characteristics and intervention protocols persists.^[6]^ These findings underscore both the clinical interest and the continuing uncertainty regarding the magnitude, consistency, and functional significance of acupuncture effects for lower-extremity recovery.

Given the ongoing burden of poststroke mobility disability and the variable strength and consistency of recommendations for acupuncture within rehabilitation guidance documents, an updated and outcome-oriented synthesis is warranted.^[7,8]^ Therefore, the present study aimed to systematically evaluate randomized controlled trials (RCTs) examining the impact of acupuncture on lower-limb motor function recovery after stroke and to quantify pooled effects on validated outcomes, including the Fugl-Meyer Assessment Lower Extremity (FMA-L), balance assessed by the Berg Balance Scale (BBS), and activities of daily living assessed by the Modified Barthel Index (MBI). By integrating evidence across international and Chinese databases without language restriction and applying contemporary risk-of-bias assessment, this systematic review and meta-analysis sought to provide a clinically interpretable synthesis to inform rehabilitation planning and to identify key sources of heterogeneity for future trial design.

## 2. Materials and methods

### 2.1. Search strategy

This systematic review and meta-analysis was conducted in accordance with the Preferred Reporting Items for Systematic Reviews and Meta-Analyses statement.^[9]^ Because this review used aggregate data from previously published studies and did not involve collection of individual patient data, formal patient consent was not required. A comprehensive electronic search was performed to identify RCTs evaluating the effects of acupuncture on lower-limb motor function recovery after stroke. The following databases were searched from inception to December 31, 2025: PubMed, Embase, Web of Science, and the Cochrane Library. No language restrictions were applied. For non-English publications, records with an English title and/or abstract were eligible for screening. The review protocol was not prospectively registered in the International Prospective Register of Systematic Reviews; however, the eligibility criteria, prespecified outcomes, and statistical approach were defined before study screening and data extraction. The absence of protocol registration is explicitly acknowledged as a transparency limitation in the Discussion.

Search terms were developed a priori based on the Population Intervention Comparator Outcomes Study design framework and combined controlled vocabulary with free-text keywords. The core concepts included stroke, acupuncture, and lower-extremity motor recovery and rehabilitation. Stroke-related terms included ischemic stroke, hemorrhagic stroke, cerebrovascular accident, and related synonyms. Acupuncture-related terms included acupuncture, manual acupuncture, electroacupuncture, scalp acupuncture, and auricular acupuncture. Lower-limb recovery terms covered lower extremity and leg function, gait and walking ability, mobility and balance, and functional recovery outcomes such as the FMA-L subscale, Functional Ambulation Category, BBS, Timed Up and Go, 10-meter walk test, and 6-minute walk test. Database-specific controlled vocabulary was applied where available, and all searches used Boolean operators and field tags appropriate to each database. Where supported, validated methodological filters were applied to restrict retrieval to RCTs. To maximize retrieval, the reference lists of included studies and relevant reviews were manually screened, and forward citation tracking was performed for key eligible articles. Additional potentially relevant records were identified through supplementary searches of related literature sources.

### 2.2. Inclusion and exclusion criteria

Studies were included if they were RCTs enrolling patients with a clinical diagnosis of stroke, including ischemic or hemorrhagic stroke, with impaired lower-limb motor function. Eligible interventions comprised needle-based acupuncture modalities, including manual acupuncture and electroacupuncture, delivered alone or as an adjunct to standard rehabilitation or usual care. Comparators included sham acupuncture, no acupuncture, a waiting list, usual care, conventional rehabilitation, or the same co-interventions without acupuncture in add-on designs. Trials were required to report at least 1 quantitative outcome related to lower-extremity motor recovery, gait, balance, or mobility, such as FMA-L scores, Functional Ambulation Category, BBS, Timed Up and Go, 10-meter walk test, or 6-minute walk test.

Studies were excluded if they were nonrandomized designs, quasi-experimental studies, case series, cross-sectional studies, reviews, or animal experiments. Trials were excluded when participants did not have a stroke or when lower-limb motor recovery was not assessed. Interventions were excluded if they did not involve needle insertion, such as acupressure, moxibustion alone, cupping, massage, or non-acupuncture electrotherapy, or if acupuncture was combined with other traditional interventions without an appropriate comparator that received identical co-interventions, preventing attribution of effects to acupuncture. Studies lacking extractable outcome data, duplicative reports from the same cohort without additional usable information, or reports with irretrievable full text were also excluded.

### 2.3. Literature screening and data extraction

All records retrieved from the databases were imported into reference management software, and duplicate citations were removed before screening. Two reviewers independently screened titles and abstracts according to the predefined inclusion and exclusion criteria. Studies considered potentially eligible by either reviewer proceeded to full-text review. Full texts were then assessed independently by the same reviewers to confirm eligibility, and the reasons for exclusion at this stage were documented. Any disagreements were resolved through discussion, and a third reviewer was consulted when consensus could not be achieved. The overall study selection process was summarized in a Preferred Reporting Items for Systematic Reviews and Meta-Analyses flow diagram.

Data extraction was conducted independently by 2 reviewers using a standardized, pilot-tested extraction form. Extracted information included the first author’s name, publication year, study period when available, sample size, sex distribution and/or gender ratio, stroke course and time since onset, participant age, and details of the intervention time course. Intervention data covered acupuncture modality, treatment frequency, session duration, total number of sessions, total treatment duration, and co-interventions. Comparator characteristics were recorded, including sham procedures or the content of usual care and rehabilitation programs. Outcome data were extracted for all prespecified endpoints at each reported time point, including measures of lower-limb motor recovery, gait, balance, and mobility. For each outcome, numerical data required for pooling were collected, including means and standard deviations for continuous outcomes and event counts for dichotomous outcomes. When data were incomplete or unclear, attempts were made to contact study authors for clarification, and when multiple reports referred to the same study population, the most complete dataset was used while additional reports were consulted for supplementary methodological details.

### 2.4. Quality assessment

Two reviewers independently appraised the risk of bias for each included RCT and cross-checked their judgments. Any discrepancies were resolved through discussion until consensus was reached. Risk of bias was assessed using the Cochrane Risk of Bias tool for randomized trials version 2.0 (RoB 2.0). Each trial was evaluated across the following domains: bias arising from the randomization process, bias due to deviations from intended interventions, bias due to missing outcome data, bias in measurement of the outcome, and bias in selection of the reported result. An overall risk-of-bias judgment for each study was derived in accordance with the RoB 2.0 guidance, classifying studies as low risk of bias, some concerns, or high risk of bias.^[10]^ The certainty of evidence was assessed for the main pooled outcomes (FMA-L, BBS, and MBI) using the Grading of Recommendations Assessment, Development and Evaluation (GRADE) approach. The certainty rating started at high for RCTs and was downgraded, where appropriate, for risk of bias, inconsistency, indirectness, imprecision, and publication bias. Final certainty was categorized as high, moderate, low, or very low.

### 2.5. Statistical analysis

All meta-analyses were performed using Stata version 18.0 (StataCorp LLC). Continuous outcomes were synthesized as standardized mean differences (SMDs) with Hedges *g* correction and corresponding 95% confidence intervals (CIs) to account for differences in measurement scales across trials. When trials reported outcomes as change-from-baseline or post-intervention values, data were extracted as reported and pooled using a consistent direction of effect so that positive SMDs indicated greater improvement in the acupuncture group. Between-study heterogeneity was evaluated using Cochran’s *Q* test and quantified with the *I*^2^ statistic. Given the anticipated clinical and methodological variability across trials, a random-effects model was used to pool effect estimates, while acknowledging that both the *Q* test and random-effects variance estimates are less stable when few studies are available. Subgroup analyses and meta-regression according to acupuncture modality, treatment duration, stroke stage, or comparator type were considered but not formally performed because each outcome included fewer than 10 studies, and several potential subgroups would have contained only 1 or 2 trials, yielding unstable and potentially misleading estimates. Therefore, potential sources of heterogeneity were explored narratively using extracted study-level characteristics. Because fewer than 10 studies contributed to each meta-analysis, formal tests for funnel-plot asymmetry, including Egger test, were not performed, and the risk of publication bias was considered qualitatively. All statistical tests were two-sided, and statistical significance was defined as *P* < .05.

## 3. Results

### 3.1. Search results and study selection

The database and registry searches identified a total of 615 records, including 585 records from bibliographic databases and 30 records from trial registers. Before screening, 450 records were removed, comprising 208 duplicate records, 153 records marked as ineligible by automation tools, and 89 records removed for other reasons. The remaining 165 records underwent title and abstract screening, of which 139 were excluded as clearly not meeting the eligibility criteria. Consequently, 26 reports were sought for full-text retrieval; 3 reports could not be obtained. The full texts of 23 reports were assessed for eligibility. After full-text evaluation, 17 reports were excluded for predefined reasons, including reviews (n = 6), sequentially published articles from overlapping datasets (n = 3), insufficient data for extraction or quantitative synthesis (n = 3), and clinical trials without an appropriate control group (n = 5). Ultimately, 6 RCTs met all inclusion criteria and were included in the systematic review and meta-analysis.^[11–16]^

### 3.2. Study characteristics

This meta-analysis included 6 RCTs. All eligible trials were published between 2013 and 2022. Across studies, sample sizes ranged from 57 to 142 participants per trial, with broadly comparable sex distributions and mean ages, indicating similar baseline demographic profiles among the included populations. The intervention protocols were delivered over a treatment course of 4 to 8 weeks, although the specific acupuncture modalities and session schedules varied across trials. Importantly, the control conditions were clinically heterogeneous, including usual care, conventional rehabilitation, sham acupuncture, and add-on designs in which both groups received comparable rehabilitation, but only the experimental group received acupuncture. The acupuncture protocols also differed in modality, point selection, stimulation parameters, treatment frequency, session duration, and total treatment dose. These differences were considered clinically relevant and were used to inform the narrative interpretation of heterogeneity. Outcome assessment focused on lower-limb motor recovery and related functional domains. The primary outcome measures commonly reported included the FMA-L to quantify lower-extremity motor impairment and the BBS to evaluate balance performance. Additional functional endpoints were reported in some studies but were less consistently available for pooling.

### 3.3. Risk of bias assessment results

Using the Cochrane RoB 2.0 tool, the included randomized trials were judged to have an overall risk-of-bias profile that was predominantly low. Most studies showed low risk in key domains related to the randomization process and outcome measurement, suggesting that major selection and detection biases were generally limited. However, several domain-specific concerns were identified. A small number of trials presented a high risk of bias for incomplete outcome data, indicating potential attrition bias due to missing data that were not adequately handled. In addition, occasional high-risk judgments were observed for the blinding of participants and personnel, reflecting the practical challenges of masking in acupuncture trials and the possibility of performance bias. Selective reporting was rated as high risk in 1 study, and isolated concerns were noted regarding allocation concealment and other sources of bias. Overall, these issues were not pervasive but warrant cautious interpretation of pooled estimates (Fig. 1).

**Figure 1.**
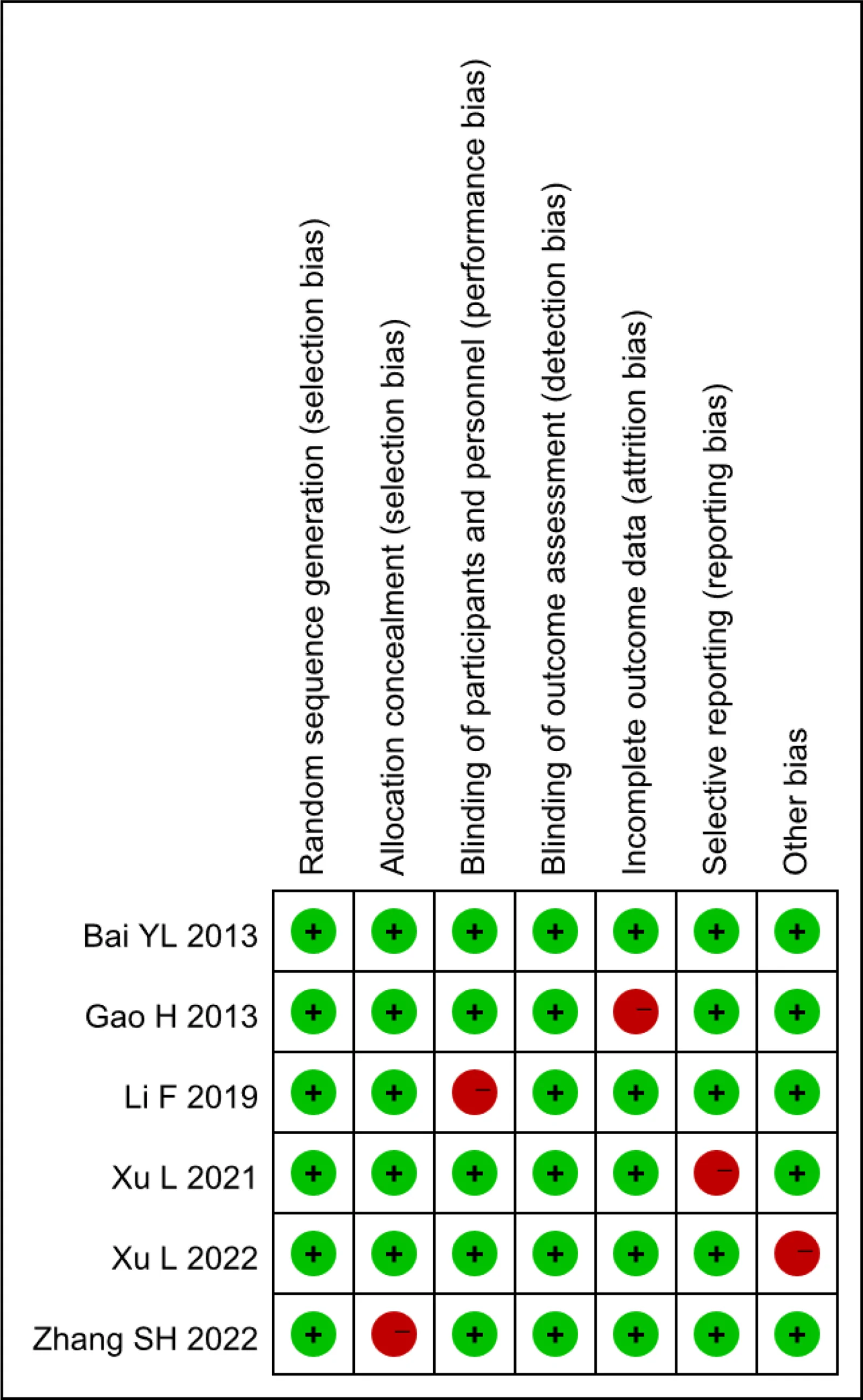
Risk-of-bias summary using the Cochrane RoB 2.0 tool across included randomized controlled trials. RoB 2.0 = Cochrane Risk of Bias tool for randomized trials version 2.0.

### 3.4. Effects of acupuncture on lower-extremity motor function

Six RCTs reported the FMA-L as an outcome for poststroke lower-limb motor recovery and were therefore eligible for quantitative synthesis. Before pooling, between-study heterogeneity was examined and indicated moderate inconsistency across trials (*I*^2^ = 53.9%, *P* = .055). In view of this heterogeneity and the anticipated clinical and methodological variability among acupuncture protocols, treatment duration, and participant characteristics, a random-effects model was applied to generate a conservative summary estimate. The pooled analysis demonstrated that acupuncture was associated with a statistically significant improvement in lower-extremity motor function compared with the control interventions. Specifically, the SMD favored acupuncture (SMD = 0.51, 95% CI = 0.32–0.71), indicating a moderate effect size in terms of FMA-L improvement (Fig. 2).

**Figure 2.**
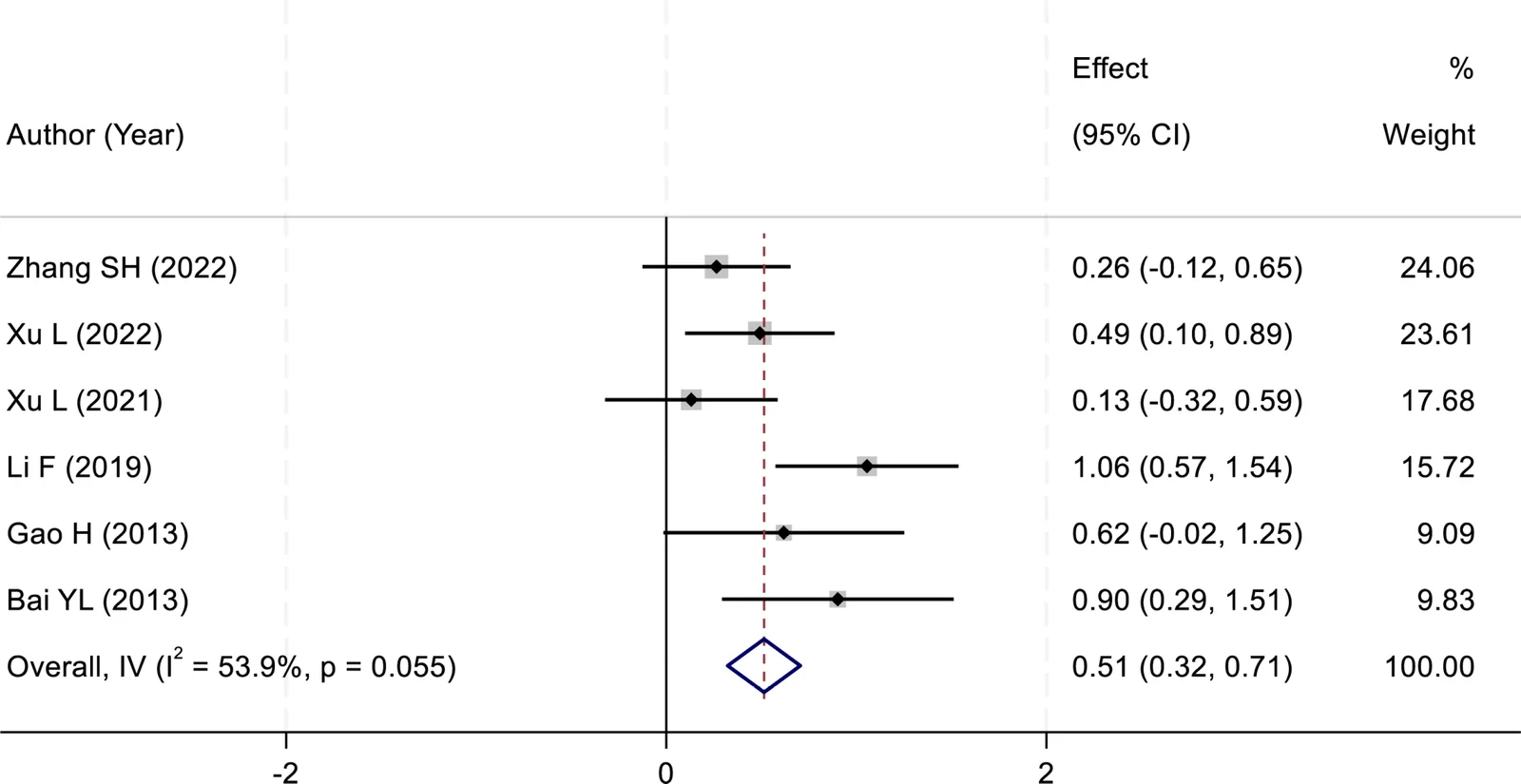
Forest plot showing the pooled effect of acupuncture on lower-extremity motor function measured by the Fugl-Meyer Assessment Lower Extremity. CI = confidence interval.

### 3.5. Results summary for balance and activities of daily living

Under a random-effects model, acupuncture was associated with a statistically significant improvement in balance compared with control interventions (SMD = 0.96, 95% CI = 0.35–1.56; *P* = .002). Heterogeneity was substantial (*I*^2^ = 91.5%, *Q* test *P* < .001), indicating considerable variability in effect magnitude across trials. Accordingly, the pooled effect should be interpreted with caution, and the observed inconsistency suggests differences in control interventions and co-intervention intensity across studies. The pooled random-effects estimate indicated a small benefit favoring acupuncture, but the CI crossed the null, and the overall effect was not statistically significant (SMD = 0.26, 95% CI = 0.16–0.68; *P* = .226). Between-study heterogeneity was high (*I*^2^ = 86.3%, *Q* test *P* < .001), implying substantial differences among trials and reduced certainty regarding the average effect on activities of daily living (Table 1).

**Table 1:** Random-effects meta-analysis results for balance and activities of daily living outcomes.

Outcome	Studies	Pooled SMD (95% CI)	*Z* test *P* value	Heterogeneity (*I*^2^)	*Q* test *P* value
BBS	4	0.96 (0.35 to 1.56)	.002	91.5%	<.001
MBI	5	0.26 (−0.16 to 0.68)	.226	86.3%	<.001
FMA-L	6	0.51 (0.32 to 0.71)	<.001	53.9%	.055

BBS = Berg Balance Scale, CI = confidence interval, FMA-L = Fugl-Meyer Assessment Lower Extremity, MBI = Modified Barthel Index, SMD = standardized mean difference.

### 3.6. Publication bias assessment results

Because fewer than 10 studies were included in each outcome-specific meta-analysis, formal statistical tests for funnel-plot asymmetry, including Egger regression test, were not performed. Therefore, the absence of publication bias cannot be concluded statistically. Instead, we considered the possibility of missing evidence qualitatively in light of the comprehensive database search, trial register search, manual reference screening, and the small overall evidence base. The pooled estimates should therefore be interpreted cautiously.

### 3.7. Certainty of evidence

Using GRADE, the certainty of evidence was rated as low for FMA-L and very low for BBS and MBI. The FMA-L evidence was downgraded mainly for imprecision related to the small number of included trials and for moderate inconsistency. The BBS and MBI evidence was downgraded for serious inconsistency, imprecision, and concerns related to heterogeneous acupuncture protocols and control conditions. These ratings indicate that the true effects, particularly for balance and activities of daily living, may differ substantially from the pooled estimates (Table 2).

**Table 2:** GRADE certainty of evidence for the main outcomes.

Outcome	Effect estimate	Certainty of evidence	Main reasons for downgrading	Plain-language interpretation
FMA-L	SMD = 0.51 (95% CI = 0.32 to 0.71)	Low	Small number of RCTs; moderate heterogeneity; possible imprecision	Current evidence suggests that acupuncture may improve lower-limb motor impairment after stroke, although further high-quality trials may change the estimate.
BBS	SMD = 0.96 (95% CI = 0.35 to 1.56)	Very low	Very high heterogeneity; small evidence base; heterogeneous controls and acupuncture protocols	Acupuncture may improve balance, but the magnitude of benefit is highly uncertain because results varied substantially across studies.
MBI	SMD = 0.26 (95% CI = −0.16 to 0.68)	Very low	Confidence interval crosses the null; very high heterogeneity; heterogeneous interventions and controls	Current evidence does not confirm a clear benefit of acupuncture on activities of daily living.

BBS = Berg Balance Scale, CI = confidence interval, FMA-L = Fugl-Meyer Assessment Lower Extremity, GRADE = Grading of Recommendations Assessment, Development and Evaluation, MBI = Modified Barthel Index, RCT = randomized controlled trial, SMD = standardized mean difference.

## 4. Discussion

This systematic review and meta-analysis synthesized evidence from 6 RCTs evaluating acupuncture for poststroke lower-extremity recovery. The pooled estimate for lower-limb motor impairment indicated that acupuncture was associated with a statistically significant improvement in FMA-L, with a moderate standardized effect size and moderate heterogeneity. However, because the evidence base was small and the GRADE certainty for FMA-L was low, this finding should be interpreted as suggesting a possible incremental impairment-level benefit rather than providing definitive proof of clinical effectiveness. Balance outcomes measured by BBS showed a larger pooled effect but substantial heterogeneity (*I*^2^ > 90%) and very low certainty, indicating that the magnitude of benefit was inconsistent across trials and that the pooled summary should be interpreted as an average across materially different comparisons. For activities of daily living, the pooled MBI estimate did not reach statistical significance and was accompanied by high heterogeneity, and was rated as very low certainty, implying that functional transfer from impairment-level improvement to broader independence outcomes remains uncertain under current evidence conditions.

Our findings are directionally consistent with a recent meta-analysis focused on lower-limb motor dysfunction after stroke, which reported improvements in FMA lower-extremity scores and BBS with acupuncture compared with conventional treatment, while also noting substantial heterogeneity for several endpoints.^[17]^ The concordance across syntheses supports the inference that acupuncture may have measurable effects on impairment-level and balance-related outcomes in some trial contexts, but that effect sizes vary across studies. Evidence from a 2024 network meta-analysis comparing acupuncture modalities for poststroke lower-limb dysfunction suggested that body acupuncture combined with rehabilitation is among the most frequently studied approaches and may rank favorably for lower-limb outcomes, although the certainty of evidence varied and the authors emphasized the need for higher-quality RCTs.^[5]^ These observations align with the heterogeneity seen in the present synthesis, where differences in acupuncture modality and co-intervention structure are plausible contributors. Beyond impairment scales, a 2025 systematic review and meta-analysis examining gait-related parameters reported that acupuncture added to rehabilitation improved stride and step length, cadence, and walking speed compared with rehabilitation alone, but heterogeneity was also prominent for several gait metrics.^[6]^ Collectively, recent syntheses suggest a recurring pattern: outcomes closely linked to motor performance and balance may show benefit, whereas cross-study inconsistency remains common. Finally, contemporary guideline-oriented evidence mapping indicates that recommendations for acupuncture in stroke rehabilitation differ by target symptom and by guideline, with stronger agreement in some poststroke indications than others, reflecting variability in the underlying evidence base.^[18]^

The clinical relevance of the pooled findings requires cautious interpretation because SMDs are scale-free, and the included trials did not consistently provide sufficient raw mean-difference data to support reliable back-translation into original scale units. An SMD of 0.51 for FMA-L corresponds to an approximate half-standard-deviation between-group advantage, which is conventionally considered a moderate effect and may be clinically meaningful for impairment-level lower-limb motor control. However, it should not be interpreted as a fixed point change on the 0 to 34 FMA-L scale without outcome-specific standard deviations and minimal clinically important difference estimates from comparable stroke populations. For BBS, the pooled effect favored acupuncture, but the very high heterogeneity and heterogeneous control conditions make the magnitude of balance improvement uncertain. For MBI, the CI crossed the null, and activities of daily living are influenced by multiple factors beyond lower-limb motor control, including upper-limb function, cognition, mood, neglect, caregiver support, environmental adaptations, and the intensity of multidisciplinary rehabilitation. Therefore, the current evidence suggests possible impairment-level benefit, but it does not yet establish a consistent or clinically quantifiable improvement in global functional independence.

A moderate pooled improvement in FMA-L may be clinically relevant because FMA-L captures impairment-level motor control in the lower extremity, which is a prerequisite for gait and task-specific training. The dissociation between clearer signals for impairment and balance outcomes versus nonsignificant pooled effects on MBI may reflect the multifactorial determinants of activities of daily living after stroke. MBI is influenced by upper-limb function, cognition, mood, neglect, caregiver support, environmental adaptations, and the intensity of multidisciplinary rehabilitation; therefore, an intervention primarily targeting neuromotor control may not consistently translate into measurable gains in global independence within short follow-up periods. This interpretation is compatible with recent literature noting that acupuncture trials frequently vary in co-interventions and that functional outcomes may require longer time horizons and standardized rehabilitation exposure to detect incremental effects.^[19,20]^ Mechanistically, several pathways have been proposed for acupuncture-related recovery effects in stroke rehabilitation, including modulation of corticospinal excitability, sensorimotor integration, spasticity-related neuromuscular control, and neurotrophic signaling relevant to synaptic plasticity. While these mechanisms cannot be confirmed from the present clinical synthesis, they provide biologically plausible explanations for why impairment-level measures and balance might respond more consistently than broad disability indices. Contemporary reviews of acupuncture for poststroke lower-limb dysfunction summarize accumulating neurophysiological and clinical observations consistent with these pathways, although the strength of evidence differs across outcomes and study designs.^[21]^

The marked heterogeneity for BBS and MBI suggests that between-study differences are substantial and likely multifactorial. First, comparator type is a major candidate source of heterogeneity. Trials comparing acupuncture plus rehabilitation versus rehabilitation alone estimate an “add-on” effect, whereas trials using sham acupuncture or usual care estimate different causal contrasts. Pooling these different contrasts may introduce structural heterogeneity and complicate interpretation of the combined effect size.^[17]^ Second, acupuncture exposure differed across studies in modality, point selection, stimulation parameters, session frequency, treatment duration, and total dose. Such protocol variability may affect both reproducibility and treatment response. Third, participant-level characteristics may have varied across trials, including time since stroke onset, baseline impairment severity, stroke subtype, rehabilitation dose, and concomitant therapies, each of which can modify responsiveness to interventions targeting neuroplasticity. Fourth, outcome timing and measurement procedures can introduce variability; short-term posttreatment assessments may capture transient performance changes, whereas longer follow-up may reflect consolidation through practice and rehabilitation dose. Fifth, methodological limitations can contribute, including incomplete blinding, differential co-intervention intensity, and missing data handling, which can inflate or attenuate effects and increase dispersion. Given the limited number of studies per outcome, formal subgroup analyses and meta-regression were not expected to provide stable estimates; therefore, these sources of heterogeneity were explored qualitatively rather than statistically.

This review has several strengths. The search strategy covered major international and Chinese databases without language restriction, increasing capture of relevant RCT evidence. Risk of bias was assessed using RoB 2.0 with independent review processes, supporting transparent appraisal. The use of SMD facilitated synthesis across trials using related, but not identical, measurement scales, and random-effects pooling provided conservative estimates in the presence of heterogeneity.

Several limitations should be considered when interpreting the conclusions. First, only 6 RCTs were eligible, which reduces statistical power, widens uncertainty, and limits the stability of pooled estimates. The small evidence base also precluded reliable subgroup analyses, sensitivity analyses, or meta-regression to identify sources of heterogeneity. Second, heterogeneity was substantial for BBS and MBI, reflecting variability in comparators, acupuncture protocols, co-interventions, stroke stage, baseline severity, and follow-up timing; consequently, a single pooled estimate may not represent a uniform treatment effect. Third, the review was not prospectively registered in the International Prospective Register of Systematic Reviews, which limits transparency regarding whether all review methods were fully prespecified and may increase concern about selective methodological or outcome decisions. Fourth, GRADE assessment indicated low certainty for FMA-L and very low certainty for BBS and MBI, mainly because of inconsistency, imprecision, and the small number of trials. Fifth, when fewer than 10 studies contribute to an outcome, assessments of small-study effects and publication bias have limited interpretability; therefore, no firm conclusion can be drawn regarding the absence of publication bias. Finally, generalizability may be limited if most trials were conducted in settings with established acupuncture practice patterns and may not reflect resource availability or standard care pathways elsewhere.

## 5. Conclusion

This systematic review and meta-analysis indicates that acupuncture may be associated with a low-certainty, moderate standardized improvement in poststroke lower-extremity motor impairment as measured by FMA-L. Balance outcomes favored acupuncture, but the evidence was very uncertain because of substantial heterogeneity. No statistically significant improvement was demonstrated for activities of daily living as measured by MBI. Future studies should be larger, prospectively registered, adequately blinded where feasible, and should use standardized acupuncture protocols, comparable rehabilitation co-interventions, prespecified clinically meaningful outcomes, and longer follow-up to clarify whether impairment-level improvements translate into durable functional gains.

## Author contributions

**Conceptualization:** Yaheng Zhao, Xiaochuan Zhu, Lei Xie, Dongyi Yang, Jiabin Wang, Yangyang Liu, Tao Wu, Renyi Qian.

**Data curation:** Yaheng Zhao, Xiaochuan Zhu, Lei Xie, Dongyi Yang, Jiabin Wang, Yangyang Liu, Tao Wu, Renyi Qian.

**Formal analysis:** Yaheng Zhao, Xiaochuan Zhu, Lei Xie, Dongyi Yang, Jiabin Wang, Yangyang Liu, Tao Wu, Renyi Qian.

**Funding acquisition:** Yaheng Zhao, Lei Xie, Dongyi Yang, Jiabin Wang, Yangyang Liu, Tao Wu, Renyi Qian.

**Investigation:** Yangyang Liu, Renyi Qian.

**Writing – original draft:** Renyi Qian.

**Writing – review & editing:** Renyi Qian.

## References

[1] Jia H He J Zhao L. Combination of stem cell therapy and acupuncture to treat ischemic stroke: a prospective review. Stem Cell Res Ther. 2022;13:87. 10.1186/s13287-022-02761-y PMC8896103 35241146

[2] Li H Long D Li B. A clinical study to assess the influence of acupuncture at “Wang’s Jiaji” acupoints on limb spasticity in patients in convalescent stage of ischemic stroke: study protocol for a randomized controlled trial. Trials. 2019;20:419. 10.1186/s13063-019-3464-7 PMC6621988 31291976

[3] Wang Y Lu M Liu R. Acupuncture alters brain’s dynamic functional network connectivity in stroke patients with motor dysfunction: a randomised controlled neuroimaging trial. Neural Plast. 2023;2023:8510213. 10.1155/2023/8510213 PMC10299883 37383656

[4] Zhao Z Wu J Fan M. Altered intra- and inter-network functional coupling of resting-state networks associated with motor dysfunction in stroke. Hum Brain Mapp. 2018;39:3388–97. 10.1002/hbm.24183 PMC6866540 29691945

[5] Liu Y Tang Y Wang L. Optimal acupuncture methods for lower limb motor dysfunction after stroke: a systematic review and network meta-analysis. Front Neurol. 2024;15:1415792. 10.3389/fneur.2024.1415792 PMC11270540 39055317

[6] Sun Z Sun H Yu K. A systematic review and meta-analysis of acupuncture’s impact on hemiplegic gait recovery after stroke. Complement Ther Med. 2025;91:103181. 10.1016/j.ctim.2025.103181 40288546

[7] Zhu R-R Wang J-X Pan B. Evidence-based evaluation for stroke guidelines mentioning traditional and complementary medicine rehabilitation. BMC Complement Med Ther. 2025;25:184. 10.1186/s12906-025-04916-9 PMC12090438 40390019

[8] Stroke rehabilitation: synopsis of the 2024 U.S. Department of Veterans Affairs and U.S. Department of Defense clinical practice guidelines. Ann Intern Med. 2025;178:249–68. 10.7326/ANNALS-24-02205 39832369

[9] Page MJ McKenzie JE Bossuyt PM. The PRISMA 2020 statement: an updated guideline for reporting systematic reviews. BMJ. 2021;372:n71. 10.1136/bmj.n71 PMC8005924 33782057

[10] Sterne JAC Savović J Page MJ. RoB 2: a revised tool for assessing risk of bias in randomised trials. BMJ. 2019;366:l4898. 10.1136/bmj.l4898 31462531

[11] Zhang SH Wang YL Zhang CX. Effects of interactive dynamic scalp acupuncture on motor function and gait of lower limbs after stroke: a multicenter, randomized, controlled clinical trial. Chin J Integr Med. 2022;28:483–91. 10.1007/s11655-021-3525-0 34913147

[12] Xu L Li L Du JT Tong X Lu Y Li F. Effect of acupuncture at Huatuo Jiaji (EX-B2) combined with core muscle training on motor function of lower limbs in patients with hemiplegia after stroke. Zhen Ci Yan Jiu. 2022;47:154–9. 10.13702/j.1000-0607.20210487 35218626

[13] Xu L Li F Wang M Yan XZ Du YH. Scalp acupuncture combined with suspension training for balance dysfunction in patients with stroke: a randomized controlled trial. Zhongguo Zhen Jiu. 2021;41:1308–12. 10.13703/j.0255-2930.20201217-0002 34936265

[14] Li F Sun Q Shao XM. Electroacupuncture combined with PNF on proprioception and motor function of lower limbs in stroke patients: a randomized controlled trial. Zhongguo Zhen Jiu. 2019;39:1034–40. 10.13703/j.0255-2930.2019.10.002 31621252

[15] Gao H Li X Gao X Ma B. Contralateral needling at unblocked collaterals for hemiplegia following acute ischemic stroke. Neural Regen Res. 2013;8:2914–22. 10.3969/j.issn.1673-5374.2013.31.004 PMC4146169 25206612

[16] Bai YL Li L Hu YS. Prospective, randomized controlled trial of physiotherapy and acupuncture on motor function and daily activities in patients with ischemic stroke. J Altern Complement Med. 2013;19:684–9. 10.1089/acm.2012.0578 23600965

[17] Chen P Jin X Yu D. Efficacy of acupuncture on lower limb motor dysfunction following stroke: a systematic review and meta-analysis of randomized controlled trials. PLoS One. 2025;20:e0312918. 10.1371/journal.pone.0312918 PMC12057971 40333934

[18] Wang X Xiao L Lin X Zhang F Zhang K Dong B. Efficacy and safety of acupuncture or moxibustion combined with rehabilitation therapy for post-stroke shoulder-hand syndrome: a systematic review and meta-analysis of randomized controlled trials. J Stroke Cerebrovasc Dis. 2026;35:108509. 10.1016/j.jstrokecerebrovasdis.2025.108509 41325795

[19] Zhuo Y Xu M Deng S. Efficacy and safety of dissimilar acupuncture intervention time-points in treating stroke: a systematic review and network meta-analysis. Ann Palliat Med. 2021;10:10196–212. 10.21037/apm-21-1127 34498479

[20] Tang Y Wang L He J Xu Y Huang S Fang Y. Optimal method of electrical stimulation for the treatment of upper limb dysfunction after stroke: a systematic review and Bayesian network meta-analysis of randomized controlled trials. Neuropsychiatr Dis Treat. 2021;17:2937–54. 10.2147/NDT.S332967 PMC8450164 34552328

[21] Xie W Di Z Shao W Wang A Guan L. Acupuncture for post-stroke lower limb dysfunction: clinical efficacy and neurophysiological mechanisms. J Multidiscip Healthc. 2025;18:7691–703. 10.2147/JMDH.S567206 PMC12665247 41328391

